# Acellular Dermal Matrices in Aesthetic Surgery: Clinical Applications

**DOI:** 10.1111/jocd.71043

**Published:** 2026-07-14

**Authors:** Olena Sydorchuk, Andrea T. Nguyen, Kyu‐Ho Yi

**Affiliations:** ^1^ Medical Research Inc Wonju Republic of Korea; ^2^ You and I Clinic Seoul Republic of Korea

**Keywords:** acellular dermal matrix, decellularized dermis, extracellular matrix, Juveacell

## Abstract

**Background:**

Acellular dermal matrices (ADMs) are decellularized extracellular matrix scaffolds derived from human or animal dermis and are increasingly used as adjuncts in aesthetic and cosmetic plastic surgery.

**Objective:**

This narrative review summarizes the mechanisms, clinical applications, safety considerations, and evidence limitations of ADM use in aesthetic surgery.

**Methods:**

A narrative literature review was conducted using PubMed/MEDLINE and reference screening to identify clinical reports, comparative studies, systematic reviews, society assessments, animal studies, and histologic studies relevant to ADM incorporation, remodeling, capsule formation, and aesthetic outcomes.

**Results:**

ADM applications in aesthetic surgery include facial contour camouflage, rhinoplasty soft‐tissue augmentation, lower eyelid spacer grafting, implant pocket control, implant camouflage, and selected management of capsular contracture in cosmetic breast surgery. The quality of evidence varies by indication. Facial and rhinoplasty applications are supported mainly by small case series, lower eyelid spacer grafting has comparative evidence including a prospective randomized study, and cosmetic breast applications are supported primarily by retrospective cohorts and large clinical series. Reported complications include seroma, infection, delayed integration, graft contraction, contour irregularity, extrusion, and cost‐related limitations.

**Conclusions:**

ADMs may be useful adjuncts in selected aesthetic surgery patients when applied according to a clear anatomic or biologic rationale. However, stronger long‐term comparative studies, standardized outcomes, and cost–benefit analyses are still needed.

## 1 Introduction

The use of biologic scaffolds has expanded from reconstructive wound coverage and burn care into aesthetic surgery, where contour stability, implant position, and scar behavior can strongly influence patient satisfaction. Acellular dermal matrices occupy a distinct role among biologics because they are derived from dermis, a tissue rich in collagen, elastin, and extracellular matrix architecture. Modern ADM processing aims to remove immunogenic cellular components while preserving the structural and biologic features that support host remodeling after implantation.

In aesthetic surgery, ADM use generally serves three purposes: structural reinforcement, contour camouflage, and biologic interface modulation. ADM may reinforce weak or revised soft tissues, improve coverage over implants or irregular cartilage and bone, and potentially influence fibrotic responses around breast implants. These benefits must be balanced against additional operative time, cost, and risks associated with implanted biologic material.

This review synthesizes the literature on ADM use in aesthetic and aesthetic‐adjacent surgery, with emphasis on sheet and spacer applications. Because ADM products differ in tissue source, thickness, crosslinking, sterilization, and handling characteristics, outcomes should not be generalized across all products without caution.

## 2 Methods

This manuscript was prepared as a narrative literature review of ADM use in aesthetic and aesthetic‐adjacent surgery. The review focused on clinical applications, mechanistic rationale, outcomes, complications, and practical considerations for patient selection and surgical technique.

PubMed/MEDLINE was used as the primary database. Search terms included “acellular dermal matrix,” “acellular dermis,” “decellularized dermis,” “extracellular matrix scaffold,” “biologic matrix,” “facial augmentation,” “rhinoplasty,” “lower eyelid retraction,” “spacer graft,” “breast augmentation,” “revision augmentation,” “capsular contracture,” “implant malposition,” “rippling,” and “pocket reinforcement.” Reference lists of relevant articles and reviews were also screened to identify additional studies.

Eligible studies included clinical reports, comparative studies, systematic reviews, society assessments, animal studies, and histologic studies relevant to ADM incorporation, remodeling, capsule formation, or aesthetic outcomes. Single case reports were generally excluded unless they described clinically important complications or failure mechanisms. Because of heterogeneity in ADM products, indications, surgical techniques, outcome definitions, and follow‐up duration, quantitative meta‐analysis was not performed. Findings were synthesized qualitatively by anatomic indication and level of available evidence. Human‐derived ADM products discussed in this review are manufactured from donated human tissues obtained through legally authorized tissue banks in accordance with applicable national regulations.

This review did not involve any new experiments using humans or animals. Any product photographs, schematic illustrations, or representative histologic images included in this review were reproduced or adapted from previously generated data with appropriate authorization and are presented for explanatory purposes only. As this review used previously published data only and did not involve identifiable private information, institutional review board approval and informed consent were not required.

## 3 Results

### 3.1 ADM Composition, Processing, and Biologic Integration

Dermal matrices are designed to preserve the extracellular framework of dermis, including collagen bundles, elastin, and matrix‐associated proteins, after removal of cells and cellular debris. Decellularization methods can affect residual DNA, collagen integrity, mechanical strength, and host response. Insufficient decellularization may increase immunogenicity, whereas overly aggressive processing may damage the matrix and reduce mechanical performance. After implantation, recellularization and vascular ingrowth depend on the local wound environment, contact with vascularized tissue, and absence of infection or seroma, which can separate the graft from host tissue and delay incorporation [1, 2].

After implantation, ADMs undergo inflammatory cell infiltration, fibroblast ingrowth, neovascularization, and gradual matrix turnover. This remodeling is desirable when it produces pliable vascularized tissue that reinforces thin or weakened soft‐tissue. However, thinning, partial resorption, or contraction may occur when ADM is placed under tension, poorly immobilized, poorly vascularized, or exposed to contamination. Surgeons should therefore regard ADM as a biologic scaffold that changes over time rather than as an inert permanent mesh.

**FIGURE 1:**
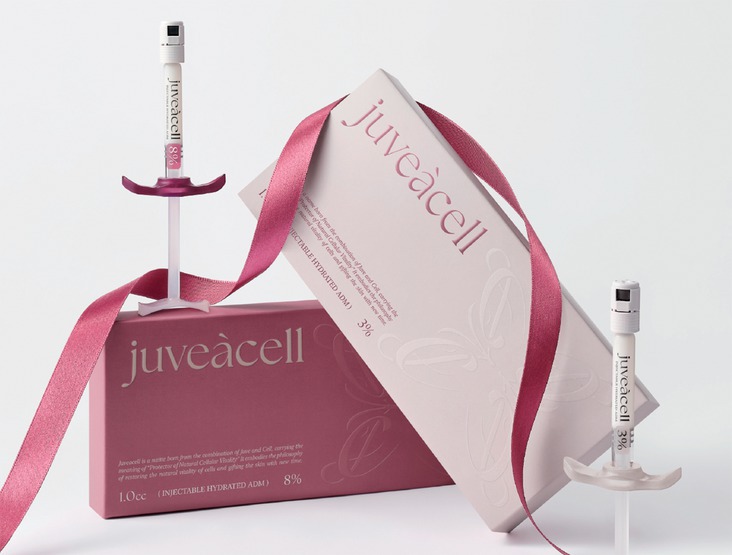
Juveàcell (VAIM, Seoul, Korea) is described in the leaflet as an ECM‐based filler/skin reconstructor made with highly biocompatible Acellular Dermal Matrix (ADM), intended to restore cellular vitality and support skin reconstruction. The ECELL supercritical CO_2_ fluid‐based process removes unnecessary components while preserving collagen and ECM essential for skin regeneration. It is positioned for natural tightening, sagging skin, fine lines, thinning skin, reduced elasticity, uneven texture/tone, pore minimization, pigmentation improvement, strengthened skin barrier, and wrinkle improvement.

The biologic sequence of ADM integration within the dermal environment is illustrated in Figure 1.

### 3.2 Mechanistic Rationale in Implant Interfaces and Capsular Biology

Capsular contracture is a multifactorial fibrotic complication influenced by bacterial contamination and biofilm, inflammation, hematoma or seroma, implant characteristics, pocket location, and patient factors. Current treatment strategies aim to address both the mechanical pocket environment and biologic drivers of fibrosis [3, 4, 5, 6, 7].

A central hypothesis for ADM use at the implant interface is that it alters the tissue response adjacent to the implant, producing a capsule that is less densely collagenized and less contractile. Experimental and translational studies support this concept. In a primate model, partial implant coverage with AlloDerm prevented capsule formation in areas where ADM contacted the implant at 10 weeks, with reduced myofibroblast staining compared with control implants. In humans undergoing implant‐based breast reconstruction, histopathologic comparison of integrated ADM with native capsule showed significantly reduced inflammatory and fibrotic parameters in the ADM region, suggesting that the biologic interface differs from conventional capsule tissue. In a rat model evaluating radiation‐related capsule formation, AlloDerm wrapping reduced radiation‐associated inflammation and pseudoepithelium formation, consistent with an interface‐modulating effect that could be clinically relevant in high‐risk fibrotic environments [8, 9, 10].

While these mechanistic studies are not definitive proof of long‐term clinical benefit in cosmetic augmentation, they provide biologic plausibility for the clinical reports in which ADM is used to treat recurrent capsular contracture or stabilize revision pockets. This plausibility is strongest when ADM is positioned to directly influence the implant–tissue interface over a meaningful surface area and when the clinical outcome of interest is plausibly linked to local fibrosis and contractile behavior [8, 9, 10, 11, 12, 13, 14, 15, 16].

**FIGURE 2:**
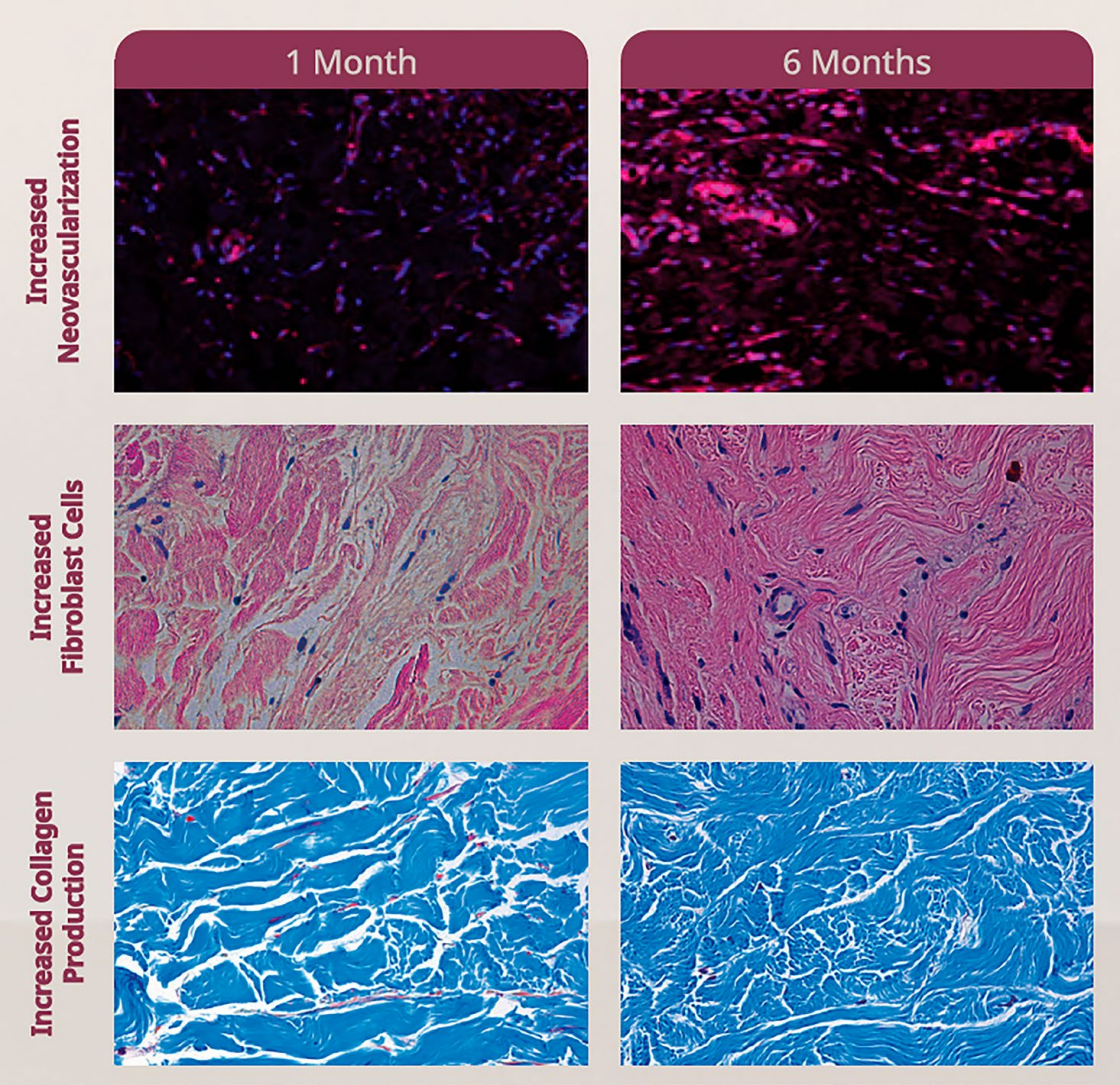
Histologic changes after injection of Juveàcell, an injectable hydrated acellular dermal matrix processed using ECELL supercritical CO_2_ technology. Representative rat‐tissue images demonstrate progressive tissue remodeling at 1 and 6 months after ADM injection. Compared with the 1‐month findings, the 6‐month specimens show more prominent neovascularization, increased fibroblast cellularity, and denser collagen deposition, suggesting sustained extracellular‐matrix remodeling and regenerative tissue response over time. Juveàcell is designed using an ECELL supercritical CO_2_ fluid‐based process that removes unnecessary cellular components while preserving collagen and ECM elements considered important for skin regeneration. Data adapted from a previously published animal study (Animal Ethics Approval No. BA‐2110‐329‐008‐01).

A schematic representation of scaffold‐mediated modulation at the implant–tissue interface is illustrated in Figure 2.

### 3.3 Facial Soft‐Tissue Augmentation and Contour Correction

The earliest aesthetic uses of ADM in the craniofacial region focused on soft‐tissue augmentation and contour smoothing. In clinical observational studies, acellular dermal grafts were evaluated in both sheet and injectable forms for facial soft‐tissue augmentation with histologic assessment and clinical follow‐up, demonstrating feasibility and providing early safety signals. These reports established key practical principles that remain relevant: the importance of appropriate graft thickness for the defect, the tendency of biologic grafts to remodel over time, and the need to minimize shear and dead space to facilitate incorporation [17, 18].

In modern facial aesthetic practice, ADM is generally used as a conservative adjunct rather than as a primary volume replacement. It may be useful for modest augmentation, contour camouflage, or soft‐tissue thickening in patients with thin tissues, prior surgery, or limited autologous options. Because contemporary aesthetic evaluation is influenced by facial proportions, aging patterns, filler‐based volumization, and patient expectations, ADM should be selected according to anatomic need rather than as a general substitute for fillers or fat grafting [17, 18, 19, 20, 21, 22, 23, 24].

### 3.4 Rhinoplasty Applications

Rhinoplasty is an archetypal setting in which small surface irregularities can be highly visible and difficult to treat, particularly in thin‐skinned patients and revision cases. ADM has been used as a camouflage layer over dorsal irregularities, as a smooth interface over cartilage graft edges, and as a soft‐tissue thickening adjunct in patients with compromised soft‐tissue envelopes. Clinical reports describe using AlloDerm to address dorsal nasal irregularities and contour issues, supporting its role as a biologic “filler” or “blanket” rather than as a rigid structural graft [25, 26, 27].

Earlier experiences also explored AlloDerm in secondary rhinoplasty settings, framing it as a method to “waste not, want not” by improving dorsal contour without harvesting additional autologous material in already‐operated noses. Across these reports, common themes include the importance of appropriate thickness selection, careful fixation to reduce migration, and recognition that partial resorption can occur, particularly when the graft is thin or poorly supported. Surgeons have generally emphasized ADM as a camouflage adjunct rather than a substitute for autologous cartilage when true structural support or projection is required [25, 26, 27].

The evidence base in rhinoplasty remains largely limited to case series and expert technique reports. ADM may reduce palpability and visibility of underlying grafts or surface irregularities, especially in thin‐skinned or revision patients. However, long‐term volume retention is variable, and ADM should be considered a camouflage adjunct rather than a substitute for structural cartilage support [25, 26, 27].

### 3.5 Lower Eyelid Retraction Repair and Periorbital Spacer Grafting

Lower eyelid retraction repair often requires release of retractor scarring and the interposition of a spacer graft to restore lid height and contour. Traditional spacer options include hard palate mucosa and autologous cartilage, each of which has advantages but also donor‐site morbidity or limitations in availability and shaping. The periorbital use of bioengineered acellular dermal matrix (BADM) spacer grafts has therefore been investigated as an alternative that avoids a second surgical site [28, 29, 30].

An American Academy of Ophthalmology (AAO) technology assessment reviewed the literature on BADM spacer grafts for lower eyelid retraction repair and concluded that available studies were mostly level II–III with variable endpoints and short follow‐up; reported surgeon‐assessed success rates were generally high, but long‐term safety and durability remained uncertain. This is an important point for aesthetic practice because lower eyelid surgery outcomes can change with time as scarring evolves and midface support changes, making long‐term stability a key outcome [29].

Higher‐quality comparative evidence is available in this domain than in many other aesthetic ADM applications. A prospective randomized comparison of lower eyelid retraction repair evaluated spacer graft options including autologous auricular cartilage, bovine acellular dermal matrix (SurgiMend), and porcine acellular dermal matrix (ENDURAGen), providing head‐to‐head data on a clinically meaningful endpoint. Autogenous hard palate mucosa remains a widely cited comparator; a long‐term clinical series in the British Journal of Ophthalmology described outcomes and donor‐site morbidity, illustrating both the durability of autologous mucosal spacers and the practical disadvantages that motivate interest in ADM alternatives [28].

Taken together, these data suggest that ADM spacers may be effective in selected patients, particularly when donor site avoidance is important. However, long‐term durability remains less certain than for some autologous spacer options. Patients should be counseled about possible contraction, integration variability, and late regression, especially in cicatricial conditions such as thyroid eye disease or post‐blepharoplasty scarring [28, 29, 30].

### 3.6 Cosmetic Breast Surgery: Implant Camouflage, Pocket Control, and Revision Strategies

ADM use in aesthetic breast surgery has expanded most visibly in revision settings, where surgeons must address implant malposition, contour irregularities, rippling, thin tissue coverage, and recurrent capsular contracture. A major early aesthetic series described using ADM in revisionary aesthetic breast surgery, reporting that ADM could be used to treat established contracture and malposition while improving implant cushioning and stabilization. Subsequent Aesthetic Surgery Journal publications and related ADM‐supported breast surgery reports further detailed revision indications, implant support, and technique concepts, reflecting broad interest in ADM as a tool for difficult implant‐based breast procedures [11, 12, 13, 14, 15, 16, 31].

One of the most influential technical concepts in revision augmentation is the idea that ADM can reinforce the lower pole and inframammary fold region, functioning as an internal support layer that reduces recurrent inferior malposition, bottoming out, or visible implant edges in thin patients. The internal “brassiere” concept has also been applied outside augmentation, including use during inferior pedicle breast reduction to reduce pseudoptosis and “star‐gazing,” demonstrating that ADM can be used as a tension‐redistributing support scaffold in breast shaping more generally [32].

Porcine‐derived non‐cross‐linked ADM products have been studied in cosmetic revision surgery as well. A retrospective cohort using porcine ADM (Strattice) in revisionary cosmetic breast augmentation reported low major complication rates and suggested utility in managing contracture, irregularities, and malposition in a challenging population. Longer‐term outcome data have also been reported for porcine ADM used with neopectoral pocket strategies, describing durability and safety in revision patients while emphasizing careful technique and appropriate patient selection [15, 16].

Despite encouraging reports, most aesthetic breast studies of ADM remain level III–IV evidence, often based on single‐surgeon retrospective series with heterogeneous revision indications. Reported complications vary across studies and may be influenced by patient selection, implant pocket characteristics, ADM handling, drain use, and contamination control. Seroma, infection, delayed healing, skin necrosis, and implant loss remain important concerns when ADM is used in breast surgery [11, 12, 13, 14, 15, 16, 33, 34].

### 3.7 Capsular Contracture: Prevention and Treatment in Aesthetic Practice

Capsular contracture remains one of the most consequential complications after augmentation and a common driver of revision surgery. Large prospective risk‐factor work and systematic reviews have quantified reoperation and contracture risk and reinforced the importance of pocket plane, implant surface, contamination control, and postoperative complications such as hematoma. Traditional surgical management has included capsulectomy or capsulotomy with implant exchange and pocket change when appropriate, but recurrence remains a concern, especially in patients with multiple prior contractures [3, 4, 5, 6, 7].

The hypothesis that ADM may reduce recurrent capsular contracture has led to selective use in high‐risk revision cases. Clinical series and algorithmic approaches suggest that ADM may be most useful in patients with recurrent contracture, bilateral disease, thin soft‐tissue coverage, or prior treatment failure. However, the evidence remains largely observational, and ADM should be used strategically rather than routinely in low‐risk primary augmentation [5, 12, 13, 14, 15, 16].

Mechanistic evidence discussed earlier supports the plausibility of this approach by demonstrating reduced capsule formation or altered inflammatory/fibrotic profiles at ADM–implant interfaces in animal models and human histologic comparisons. However, aesthetic surgeons should note that reconstruction data and augmentation data are not interchangeable: radiation exposure, mastectomy flap biology, and expander use differ meaningfully from primary cosmetic augmentation. Therefore, while ADM may be an effective adjunct in selected augmentation patients with recurrent contracture or thin tissues, definitive conclusions about prophylactic ADM use in routine primary augmentation require stronger comparative evidence [5, 8, 9, 10].

### 3.8 Safety, Complications, and Risk Mitigation Across Aesthetic Indications

Across anatomic regions, ADM implantation introduces risks related to biomaterial placement, dead space, and fluid accumulation. Seroma is a recurrent concern in breast surgery and may delay incorporation by separating the matrix from vascularized host tissue. Infection risk varies by patient and procedure type; when infection occurs, ADM removal or implant loss may be required depending on severity and timing [33, 34].

In facial and rhinoplasty applications, overt infection is less commonly emphasized, but contour‐related complications such as palpability, visible edges, partial resorption, and unpredictable remodeling can affect satisfaction. In eyelid surgery, complications described in society assessments include cyst formation, infection, chemosis, granuloma, and corneal abrasion, with the key unresolved issue being long‐term stability and the risk of regression [33, 34].

Risk mitigation depends on careful patient selection and meticulous technique. Key principles include hemostasis, contamination control, dead‐space reduction, stable ADM positioning, broad contact with vascularized tissue, and appropriate postoperative fluid management when drains are used [33, 34].

### 3.9 Cost, Value, and Evidence Limitations

Cost remains a major limitation to broader ADM use in aesthetic surgery. In selected revision cases, ADM may reduce recurrent deformity, improve contour predictability, or avoid donor site morbidity. However, most studies do not include formal cost‐effectiveness analyses or standardized patient‐reported outcome measures. Therefore, ADM use should be justified by a clear anatomic or biologic rationale rather than routine preference [35, 36].

Evidence limitations are also systematic. Many aesthetic ADM publications are retrospective with mixed indications, modest follow‐up, and evolving technique. Outcomes may depend heavily on surgeon experience and patient mix, which limits generalizability. Even in better‐studied domains such as eyelid spacer grafting, higher‐level evidence remains limited and long‐term follow‐up is not consistently reported. These limitations argue for cautious interpretation, transparent counseling, and a preference for ADM use in scenarios with a clear anatomic or biologic rationale, rather than routine use in low‐risk primary aesthetic procedures [35, 36].

## 4 Discussion

### 4.1 Interpreting ADM Outcomes by Indication

The strongest justification for ADM use in aesthetic practice arises when the clinical problem involves mechanical support failure, tissue deficiency, contour irregularity, or a high‐risk fibrotic environment. In revision breast surgery, ADM can reinforce the implant pocket, improve implant camouflage, and potentially influence capsule behavior; however, most supporting studies remain nonrandomized and may be affected by selection and reporting bias [11, 12, 13, 14, 15, 16, 32]. In facial aesthetic practice, ADM should also be considered within the broader context of facial proportion, aging, volume restoration, and patient expectations, which increasingly shape how patients define satisfactory aesthetic outcomes [19, 20, 21, 22, 23, 24].

In periorbital surgery, the evidence base is comparatively structured, including randomized data comparing spacer materials and a society technology assessment synthesizing broader outcomes. This supports the conclusion that BADM spacers can be reasonable options when autologous graft harvest is undesirable or contraindicated, but surgeons should counsel that long‐term durability is less certain than some traditional autologous options [28, 29, 30].

In facial and rhinoplasty applications, ADM functions mainly as a camouflage or soft‐tissue thickening layer. Although the evidence is mostly case‐series based, the anatomic rationale is clear: ADM can create a biologic interface between thin skin and underlying graft edges or bony‐cartilaginous irregularities. The main clinical challenge is predicting remodeling‐related volume change over time [17, 18, 19, 20, 21, 22, 23, 24, 25, 26, 27]. A summary of the clinical evidence for ADM use across major aesthetic indications is presented in Table 1.

**TABLE 1:** Summary of clinical evidence for ADM use in aesthetic indications.

Indication	Key references	Study design	Sample size	ADM type	Follow‐up	Main outcomes	Level of evidence
Facial soft‐tissue augmentation	5,6	Case series	Small cohorts	Human ADM (AlloDerm)	Short–mid term	Contour improvement; remodeling observed	IV
Rhinoplasty (dorsal camouflage, revision)	7–9	Case series	Small–moderate	Human ADM (AlloDerm)	Variable	Improved dorsal contour; partial resorption in some cases	IV
Lower eyelid retraction repair	10–12	Prospective randomized (10); Level II–III studies	Moderate	Bovine (SurgiMend), Porcine (ENDURAGen), Autologous comparators	Mid‐term	Lid height improvement; contraction variability	II–III
Cosmetic breast revision (malposition, rippling)	13–19	Retrospective cohorts	Moderate–large	Human & Porcine ADM	Mid–long term	Improved pocket control; reduced malposition recurrence	III
Capsular contracture treatment/prevention	14–18,27	Cohort, algorithmic series	Moderate	Human & Porcine ADM	Variable	Lower recurrence in selected high‐risk cases	III
Mechanistic implant interface studies	20–22	Animal + histologic human	Experimental	Human ADM	Short‐term	Reduced fibrosis markers; altered capsule characteristics	Preclinical

### 4.2 Practical Considerations for Surgical Technique and Patient Selection

Several technical principles apply across indications. ADM should be placed in stable contact with well‐vascularized tissue because floating placement may increase dead space, seroma risk, and delayed incorporation. Excessive tension should be avoided to reduce thinning, edge visibility, or contraction. In breast surgery, meticulous contamination control is particularly important because biofilm‐related mechanisms are implicated in capsular contracture and implant infection [33, 34].

Patient selection should consider factors that increase complication risk or reduce incorporation reliability, including smoking, poorly controlled diabetes, immunosuppression, prior radiation, and prior seroma or infection. In cosmetic practice, ADM should be selected when its expected benefit justifies additional cost and morbidity compared with alternatives such as pocket change, implant exchange, fat grafting, or autologous tissue grafting [33, 34].

### 4.3 Future Directions

Future studies should use standardized outcome reporting, validated patient reported outcome measures, and objective measurements of contour, eyelid position, implant position, and recurrence. Long‐term follow‐up is especially important because ADM remodeling, scarring, graft contraction, and recurrent malposition may develop years after surgery. Comparative studies should also distinguish among ADM product classes, including human, porcine, and bovine matrices; crosslinked and non‐crosslinked products; and different sterilization and processing methods [35, 36]. Key characteristics of commonly used ADM products in aesthetic surgery are summarized in Table 2.

**TABLE 2:** Key characteristics of ADM products used in aesthetic surgery.

Product	Tissue source	Crosslinked	Typical use	Reported advantages	Reported limitations
AlloDerm	Human cadaveric dermis	Non‐crosslinked	Rhinoplasty, breast revision	Good integration, pliability	Variable thickness; cost
Strattice	Porcine dermis	Non‐crosslinked	Breast revision, pocket reinforcement	Strong lower‐pole support	Seroma risk
SurgiMend	Bovine dermis	Non‐crosslinked	Eyelid spacer graft	Alternative to autologous graft	Limited long‐term eyelid data
Enduragen	Porcine dermis	Crosslinked	Eyelid spacer	Increased durability	Potential reduced remodeling

## 5 Conclusion

Acellular dermal matrices are biologically active scaffolds that may reinforce soft‐tissue, improve contour camouflage, stabilize revision implant pockets, and modulate fibrotic capsule behavior in selected aesthetic surgery patients. The most consistent roles include revision breast pocket control, implant camouflage, recurrent capsular contracture management, lower eyelid spacer grafting, and selected contour smoothing in rhinoplasty or facial augmentation. However, evidence quality varies substantially by indication, and many aesthetic applications remain supported mainly by retrospective series. Complications, cost, product heterogeneity, and uncertain long‐term durability remain important limitations. Current best practice is selective, anatomy‐driven ADM use with meticulous technique and realistic patient counseling [35, 36].

## Author Contributions

All authors have read and approved the final manuscript. Conceptualization: K.‐H.Y., O.S. Literature review and data curation: O.S., A.T.N., K.‐H.Y. Writing – original draft: O.S., K.‐H.Y. Writing – review and editing: K.‐H.Y., O.S., A.T.N. Supervision: K.‐H.Y.

## Funding

The authors received no external funding for this work.

## Ethics Statement

The authors have nothing to report.

## Consent

The authors have nothing to report.

## Conflicts of Interest

The authors declare no conflicts of interest.

## Data Availability

Data sharing not applicable to this article as no datasets were generated or analysed during the current study.
